# Chronic pancreatitis: review and update of etiology, risk factors, and management

**DOI:** 10.12688/f1000research.12852.1

**Published:** 2018-05-17

**Authors:** Angela Pham, Christopher Forsmark

**Affiliations:** 1 Division of Gastroenterology, Hepatology and Nutrition, University of Florida, Gainesville, Florida, USA

**Keywords:** Pancreatitis, Chronic, fibrosis

## Abstract

Chronic pancreatitis is a syndrome involving inflammation, fibrosis, and loss of acinar and islet cells which can manifest in unrelenting abdominal pain, malnutrition, and exocrine and endocrine insufficiency. The Toxic-Metabolic, Idiopathic, Genetic, Autoimmune, Recurrent and Severe Acute Pancreatitis, Obstructive (TIGAR-O) classification system categorizes known causes and factors that contribute to chronic pancreatitis. Although determining disease etiology provides a framework for focused and specific treatments, chronic pancreatitis remains a challenging condition to treat owing to the often refractory, centrally mediated pain and the lack of consensus regarding when endoscopic therapy and surgery are indicated. Further complications incurred include both exocrine and endocrine pancreatic insufficiency, pseudocyst formation, bile duct obstruction, and pancreatic cancer. Medical treatment of chronic pancreatitis involves controlling pain, addressing malnutrition via the treatment of vitamin and mineral deficiencies and recognizing the risk of osteoporosis, and administering appropriate pancreatic enzyme supplementation and diabetic agents. Cornerstones in treatment include the recognition of pancreatic exocrine insufficiency and administration of pancreatic enzyme replacement therapy, support to cease smoking and alcohol consumption, consultation with a dietitian, and a systematic follow-up to assure optimal treatment effect.

## Definition and pathogenesis

**Figure 1.**
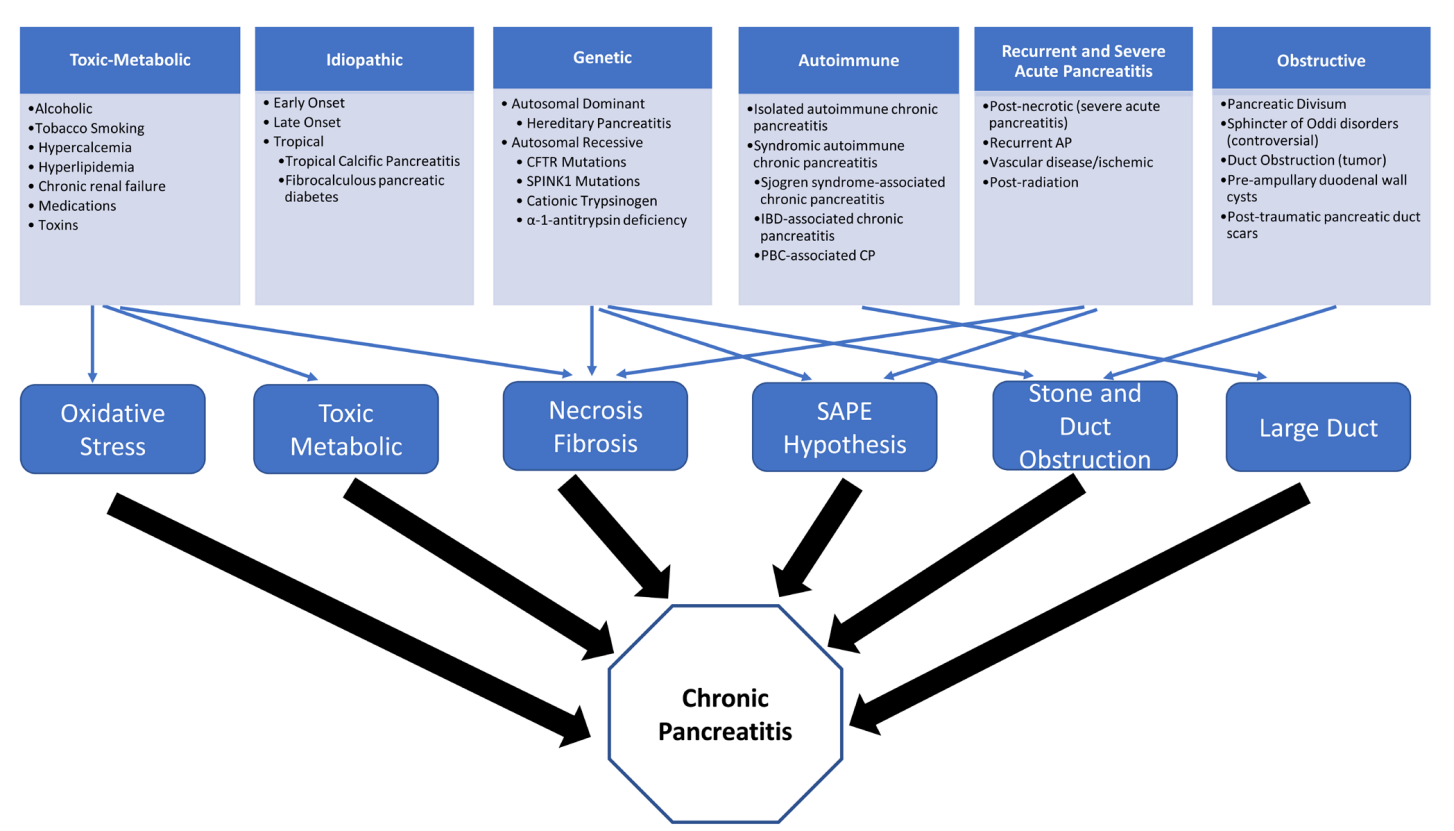
TIGAR-O risk factor classification system. AP, acute pancreatitis; CFTR, cystic fibrosis transmembrane conductance regulator; CP, chronic pancreatitis; IBD, inflammatory bowel disease; PBC, primary biliary cirrhosis; SAPE, sentinel acute pancreatitis event; SPINK1, serine protease inhibitor kazal-type 1; TIGAR-O, Toxic-Metabolic, Idiopathic, Genetic, Autoimmune, Recurrent and Severe Acute Pancreatitis, Obstructive. Adapted from ^11^.

Chronic pancreatitis (CP) is a syndrome characterized by chronic progressive pancreatic inflammation, fibrosis, and scarring, resulting in damage to and loss of exocrine (acinar), endocrine (islet cells), and ductal cells ^1^. The syndrome is commonly associated with clinical features of abdominal pain, exocrine and endocrine insufficiency, secondary pancreatic cancer, and other complications. It is accepted that inflammation-led fibrosis culminates in CP ^2^. Although acute pancreatitis (AP) and CP were believed to be distinct entities ^3^, a wealth of data support that AP, recurrent AP (RAP), and CP represent a disease continuum ^4, 5^. The etiology of CP has traditionally been classified as alcohol, hereditary, obstructive, hyperlipidemia, and idiopathic. Recent evidence supports the notion that, in most patients, more than one “etiology” is present. The TIGAR-O classification system ( Figure 1) is grouped by risk modifiers, not etiologies, that may interact to produce pancreatic disease: toxic-metabolic, idiopathic, genetic, autoimmune, recurrent and severe AP-associated CP, and obstructive etiologic factors ^6^. The development of this classification system was based on the principle that an individual’s risk of developing CP is decided by one or more risk factors ^7^. A two-hit hypothesis model can be used to outline the pathogenesis of CP ^8^: in the setting of pre-existing AP risk factors (genetic, metabolic, and environmental), an initial first episode of AP (first hit) initiates or activates the immune system, followed by complete recovery, or pathologically by progression towards CP. This cascade of steps toward CP is triggered, provided that there is ongoing damage to the pancreas via oxidative stress or repeated episodes of acute inflammation, which may or may not be clinically apparent ^4^. Collectively, this sequence has been coined the sentinel AP event (SAPE) hypothesis ^9, 10^. Overall, approximately 20% of patients with AP have a recurrence and 36% of RAP patients go on to develop CP ^4^. Here we will review further the myriad risk factors that contribute to the progression to end-stage CP and also cover the current treatment modalities for CP.

## Risk factors

### Alcohol

The pathogenesis of alcoholic pancreatitis is poorly understood, but it is thought that chronic alcohol consumption sensitizes the acinar cell to injury by interfering with mechanisms that protect against endoplasmic reticulum stress ^11^. CP is not merely the alcohol injury but a complex chronic inflammatory disorder that is linked to genetic, metabolic, and environmental factors ^12^. Alcoholic CP may initially present as a clinical episode of AP, and the development of CP is not inevitable in patients with alcoholic AP ^13, 14^. In one large natural history study, the authors investigated an association between alcohol consumption and CP in 540 patients and 695 controls ^15^. Analysis revealed a significant association between alcohol and CP only with the consumption of five or more alcoholic drinks per day, which suggests a threshold level of drinking ^15^. Ultimately, the risk of pancreatitis, even among individuals who drink heavily, is very low, estimated to be approximately 2–3% with the consumption of approximately five alcoholic drinks per day ^16^. Recently, genetic variants in the *CLDN2* gene loci have been identified that influence the risk for alcohol-related pancreatitis ^17^. These findings represent an example of the complex interplay between various risk factors in CP. Alcohol increases the risk of CP in a dose-dependent manner ^1, 18^, and evidence shows that continued exposure increases chances of progression to CP ^18, 19^. Studies have shown that periodic intervention and frequent medical follow-up reduced the risk of disease recurrence after an initial attack of acute alcoholic pancreatitis during a two-year follow-up period ^20^, which is especially relevant in a primary care setting, in which a patient is more likely to see their physican rather than a specialist over a long-term period.

### Smoking

The prevalence of smoking increases with the amount of alcohol consumed ^21^. However, new data from case-controlled studies show that there is an independent association between smoking and both AP and CP ^21^. Overall, 46% of all cases of pancreatitis could be attributed to smoking ^22^. In a recent meta-analysis, the pooled risk estimate for CP was 2.5 for current smokers when compared with never smokers after adjustment for alcohol use ^21, 23^. The risk of CP in smokers is linear, and, while acting as an independent risk factor, it is also a disease modifier, with synergistic detrimental effects in conjunction with alcohol consumption ^2^. Pancreatic ischemia worsened and leukocyte infiltration increased in an animal model of both smoking and alcohol use ^24, 25^ suggesting one possible mechanism for this synergy.

## Genetic factors in pancreatitis

CP is now recognized as a complex disease with multiple associated genetic risk factors and disease modifiers ^2^. Genetic variations strongly associated with CP are those in PRSS1 (cationic trypsinogen), SPINK1 (serine protease inhibitor kazal-type 1), and CFTR (cystic fibrosis transmembrane conductance regulator) and, to a lesser extent, CTRC (chymotrypsin C) and CASR (calcium-sensing receptor) ^2^. These mutations and polymorphisms have different mechanisms and variable penetrance. The most potent mutation is in PRSS1, a gain-of-function mutation, which can cause the autosomal dominant condition of hereditary pancreatitis (HP) ^26^. HP differs from many other forms of pancreatitis in the early onset, rapid progression to end-stage CP, and a significantly increased risk of pancreatic adenocarcinoma ^26^. Mutations and polymorphisms in other genes function as risk factors and disease modifiers. Genetic testing might be considered when patients have a family history of idiopathic CP, RAP, or childhood pancreatitis, have relatives with known mutations associated with HP, are younger than 25 years old, or have RAP of uncertain etiology ^26^.

## Autoimmune pancreatitis

Celiac disease increases the risk of CP by approximately threefold ^27, 28^. The risk is also increased among patients with inflammatory bowel disease (IBD) ^29^, systemic lupus erythematosus, and other autoimmune disorders ^2^. Autoimmune pancreatitis (AIP) is a recently recognized pancreatic inflammatory disease that is further classified into two subtypes. Type 1, called lymphoplasmacytic sclerosing pancreatitis, is a systemic disease affecting the pancreas, bile ducts, kidneys, salivary glands, retroperitoneum, and other organs. It is also associated with infiltration of these organs by IgG4-bearing plasma cells, with elevations in serum levels of IgG4 ^30^. The IgG4 is not felt to be pathogenic but is useful for diagnosis. AIP type 1 is most commonly seen in middle-aged men presenting with painless obstructive jaundice, a similar presentation to the much more common pancreatic cancer. Type 2 affects only the pancreas, is called idiopathic duct-centric pancreatitis, is not associated with IgG4 ^30^, and is commonly seen in younger patients presenting with AP. Furthermore, type 2 has no biomarker and is strongly associated with IBD. Both subtypes are corticosteroid responsive; however, relapses are typical in Type I AIP and rare in Type 2 AIP ^31, 32^. Thus, maintenance therapy with either an immunomodulator or rituximab is often necessary for patients with AIP ^33^. Prior to initiating a therapeutic trial, it is essential to rule out pancreatic malignancy.

## Anatomic abnormalities and ductal obstruction

Pancreatic ductal obstruction due to inflammatory strictures, benign tumors, or malignancies leads to chronic obstructive pancreatitis upstream from the obstruction ^1, 34^. These might include pancreatic ductal adenocarcinoma, intraductal papillary mucinous neoplasm, ampullary adenoma or carcinoma, duodenal diseases (celiac or Crohn’s disease) causing ampullary scarring, ductal strictures after a severe episode of AP, pancreatic trauma, and other more controversial conditions like sphincter of Oddi dysfunction or pancreas divisum ^35^. The potential for pancreatitis due to underlying malignancy requires a careful search for cancer in those at higher risk (generally above the age of 40). As regards pancreas divisum, the larger dorsal pancreas is drained through the minor papilla, which hypothetically could cause obstruction. Pancreas divisum is common in the general population (up to 5–10%) ^36^, but CP is rare in divisum, and it would be rarer still that CP might be confined to the dorsal pancreas in patients with pancreas divisum. A higher frequency of pancreas divisum has been noted in patients with CFTR mutation-associated pancreatitis, suggesting that these two potential risk factors cosegregrate. There is very little evidence that pancreas divisum by itself causes AP or CP, but it could act synergistically with genetic factors ^35^.

## Diagnosis of chronic pancreatitis

**Figure 2.**
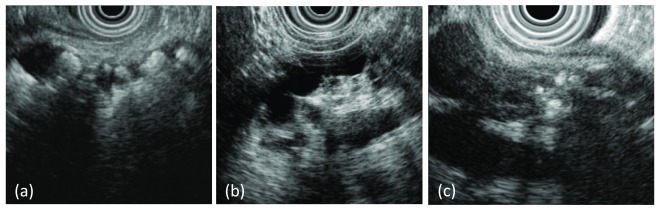
Endoscopic ultrasound images characterizing features of chronic pancreatitis. (a) Anechoic tubular structure with multiple hyperechoic structures with shadowing. Depicts a dilated main pancreatic duct with multiple calcified stones. (b) Anechoic tubular structure depicting dilated, irregular main pancreatic duct. (c) Diffuse echogenicity with hyperechoic foci with stranding. Depicts pancreatic lobularity with calcifications.

The diagnosis of CP is usually made by cross-sectional imaging, typically CT or MRI. The diagnosis in those with advanced CP is usually obvious on these studies, with pancreatic calcification, atrophy, and a dilated or irregular pancreatic duct. The addition of MRCP allows more accurate identification of pancreatic ductal abnormalities than does CT or MRI alone, particularly if the hormone secretin is administered during MRCP ^37^. The diagnosis of CP in less-advanced disease is more challenging, and a combination of endoscopic ultrasonography (EUS) ( Figure 2) and direct pancreatic function testing is utilized. Despite these techniques, early diagnosis remains difficult and often inaccurate. Interested readers are referred to a recent review of diagnostic approaches ^7^.

## Clinical features of chronic pancreatitis and their management

### Abdominal pain

The management of CP is challenging and requires a personalized approach focused on the individual patient’s main symptoms, goals, and quality of life ^38^. Most patients remain symptomatic despite therapy ^39^. Most patients with CP have abdominal pain, with a reported prevalence of 50–85% ^40, 41^. Pain in CP is multifactorial, with inflammatory and neuropathic components ^30^. The pain in the past was believed to be primarily due to obstruction of the pancreatic duct by either stricture or stone, resulting in high pressure and ischemia above the obstruction ^39^. However, duct obstruction or dilation does not actually correlate with pain in numerous studies. A second cause of pain is complications of CP. These might include a pseudocyst, bile duct or duodenal obstruction, or a secondary pancreatic cancer. In more recent studies, a third contributor to pain would be termed neuropathic. The evidence of neuropathic changes better correlates with pain in CP. These include both structural changes to the intrapancreatic nerves and functional changes in both pancreatic nociceptive neurons and spinal and central neurons involved in pain signaling and perception. There is an increased density and hypertrophy of intra-pancreatic nerves, presumably from progressive inflammation and fibrosis ^42^. Trypsin further activates these nociceptors, providing a unique (and unfortunate) trigger. These primary nociceptors have nerve bodies within the dorsal horn and communicate with second-order neurons within the spinal cord. The function of the primary nociceptive neurons and spinal neurons becomes abnormal, and a state of sensitization can develop ^39^. This leads to hyperalgesia (a magnified pain perception to a normally painful stimuli) and allodynia (pain due to normal or physiologic stimuli). In this situation, therapy directed at the pancreas or pancreatic duct is often ineffective ^30^. Medical treatment options for painful pancreatitis include avoidance of alcohol and smoking and administration of pancreatic enzymes, analgesics, antioxidants, and adjunctive agents ^39^. The majority of patients with pain will require analgesics. Long-term use of high-potency opioids in this setting is best avoided because it leads to tolerance and dependence, particularly in patients for whom drug dependence and abuse potential are already a concern from prior smoking, alcohol use, or depression. A reasonable initial approach is tramadol in dosages of 200 to 400 mg daily, although higher doses are given to some patients ^39^. Thereafter, more potent narcotics may be required, with gradual increases in potency and frequency aimed to reduce but not eliminate pain ^39^. Adjunctive pain medication such as tricyclic antidepressants, gabapentin, pregabalin, and selective serotonin-reuptake inhibitors have been used either alone or in combination with opioids with variable results ^1^. Patients treated with pregabalin (up to 300 mg twice daily) had reduced pain compared with those who were given placebo and were able to reduce opioid use ^43^. The other adjunctive agents have not been assessed in randomized trials but are reasonable additions in patients who require more potent opioids. Additional medical options for pain reduction include pancreatic enzyme replacement therapy (PERT), octreotide, and antioxidants. PERT has been widely utilized in treating pain in CP patients. Pathophysiologically, PERT is used for pain relief because it can degrade CCK-releasing factor in the duodenum and, by doing so, lowers CCK levels; additionally, through this mechanism, it reduces pain ^38^. The data supporting enzymes in this setting are limited. Large trials of antioxidants have reached different conclusions ^44^, but they are a reasonable option if other therapies fail. Little evidence supports the use of octreotide.

In some patients, non-medical options for pain are also utilized. EUS and endoscopic retrograde cholangiopancreatography (ERCP) have well-defined roles in the diagnosis and management of CP ^12^. For patients who do not respond to medical therapy, options include endoscopic therapy (ET), nerve block or neurolysis, and surgery. Many patients with poorly controlled pain, refractory to medical therapy, will not benefit from endoscopic procedures, and a detailed risk–benefit discussion and careful patient selection should precede any intervention ^1^. The best candidates for the successful treatment of CP pain with first-line ET are those with distal obstruction of the main pancreatic duct with obstructing stone or stricture in the head of the pancreas and in the early state of the disease ^45^. The current approaches of ET for CP are directed at (1) relieving obstructing pancreatic duct stones, (2) relieving pancreatic duct strictures, (3) draining pancreatic pseudocysts, (4) administering celiac plexus nerve blocks, and (5) relieving benign biliary strictures ^46^. ET comprises pancreatic and biliary sphincterotomy, stricture dilation and stenting, stone extraction, and lithotripsy ^39^. Large stones or impacted stones usually require extracorporeal shockwave lithotripsy (ESWL) or intraductal lithotripsy; these techniques apply shockwaves to break up stones ^39^. Dominant strictures in the main pancreatic duct are managed by endoscopic pancreatic duct stent placement, with current guidelines supporting the use of a single stent placed long term ^47^. Per ESGE guidelines, there is no role for ET in asymptomatic and uncomplicated CP. No study has demonstrated any benefit for ET in these patients, including for the preservation of exocrine or endocrine pancreatic insufficiency ^35, 48, 49^. Although EUS-guided celiac plexus neurolysis relieves pain in about 50% of patients, the effect lasts a maximum of a few weeks with risk for side effects such as postural hypotension and diarrhea ^45^. Hence, celiac plexus block is rarely applied in CP and is not recommended unless there is concomitant pancreatic malignancy ^50, 51^.

There is no clear consensus of opinion on whether surgery is superior to endoscopy in terms of mid-term and long-term pain relief in patients with CP ^52^. In a Cochrane review of all the randomized controlled trials to date, the main finding was that surgery achieves pain relief in a higher proportion of patients compared with endoscopic treatment for patients with obstructive CP ^53^. Additional benefits of surgery compared with endoscopy were reported as well: mainly, improved quality of life in the middle/long-term and a lower risk of developing exocrine pancreatic insufficiency ^53^. This review included two randomized controlled trials with a total of 111 participants ^53^. It should be noted that there are no sham-controlled studies. With this in mind, there is substantial evidence that some surgical interventions have a sizable “placebo effect” on patients, especially in the treatment of chronic pain ^54– 63^. A Mayo clinic systematic review studied the magnitude of the placebo effect associated with sham surgery procedures and found that there exists a large sham effect with an effect size of nearly 0.4 for improvement in subjective outcomes, including pain, disability, and quality of life ^64^. This was corroborated by a BMJ meta-analysis that demonstrated that non-specific effects accounted for 78% of the active treatment effects of surgery in chronic pain conditions ^54^. With no sham-controlled studies for surgery in painful CP, there is little evidence to estimate the effect of surgical treatment of CP. Nonetheless, current European guidelines favor early surgery over surgery at a more advanced stage of disease to achieve optimal long-term pain relief ^52^. Regardless, ET is performed first in most cases based on patient choice, with surgery most often reserved for patients whose painful symptoms do not respond well to ET. One long-term controlled trial of endoscopic pancreatic stenting to treat main pancreatic duct stricture in CP followed these patients as compared to controls for an average of 62.5 months. The endoscopically stented patients had reduced pain recurrence (15% versus 50%) and slowed progression of pancreatic exocrine insufficiency (PEI) ^65^. Once a multidisciplinary decision is made to pursue surgery, options include lateral pancreaticojejunostomy or the modified Puestow procedure, the duodenum-preserving pancreatic head resection procedures (DPPHRs: Frey, Beger, or Berne), or the Whipple procedure. The DPPHR procedures are most commonly employed for complicated CP with both a dilated pancreatic duct and an inflammatory mass in the head of the pancreas, often causing biliary or duodenal obstruction. The various techniques for DPPHR have been compared with partial pancreatoduodenectomy in several small-scale randomized trials, which have suggested superiority for DPPHR over partial pancreatoduodenectomy. However, the multicenter ChroPac trial showed no differences in quality of life after surgery between the two interventions ^66^.

The modified Puestow is least morbid and preserves the most pancreatic parenchyma but has only around 50% long-term pain relief. Total pancreatectomy is associated with a high rate of post-operative morbidity (40–50%) and results in brittle insulin-dependent diabetes that is especially challenging to manage ^67^. Total pancreatectomy is rarely indicated for the treatment of CP and is reserved only for patients who failed previous surgical interventions, who have severe pain with complete exocrine and endocrine pancreatic failure, who meet IPMN criteria for resection ^67^, or who have hereditary pancreatitis or familial pancreatic cancer as a prophylactic procedure for pancreatic cancer ^68^. Despite the relatively high morbidity and mortality of operative management of CP, nearly half of all patients with CP will eventually require some form of surgical intervention to treat chronic pain that is unmanageable via less-invasive means ^69– 71^.

### Management of exocrine insufficiency

PEI, characterized by inadequate pancreatic secretion of digestive enzymes and bicarbonate, is one of the most significant complications of CP, affecting >50% of diagnosed patients ^72^, resulting in compromised digestion, absorption, and metabolism of nutrients. Symptomatic PEI does not occur until approximately 90% of pancreatic exocrine function is lost ^73^. Exocrine insufficiency manifests as steatorrhea (often without diarrhea), weight loss, malnutrition, metabolic bone disease, and vitamin and mineral deficiency ^74^. Severe PEI tends to develop between 5 and 10 years following an initial diagnosis of CP ^39^. PEI is most common in those with CP due to alcohol, smoking, and some other etiologies including PRSS1 mutations. As a result of PEI, these populations are at risk of weight loss and malnutrition due to fat maldigestion and malabsorption ^75^. Long-term fat malabsorption may also lead to fat-soluble vitamin (A, D, E, and K) deficiencies ^75^ as well as deficiencies in calcium, magnesium, zinc, thiamine, and folic acid ^76^. It should be noted that in CP patients, osteoporosis risk is three times higher than in the general population and is apparent in even exocrine-sufficient patients ^77^. One in four CP patients have osteoporosis, and up to two-thirds have either osteoporosis or osteopenia ^78^. For this reason, bone mineral density testing should be done in all CP patients. Additionally, a baseline evaluation of nutritional status is appropriate when patients begin PERT to include weight and BMI, complete blood count, comprehensive metabolic panel, international normalized ratio, and levels of albumin, prealbumin, carotene, and vitamin D.

At present, measurement of fecal elastase is the most popular test to evaluate PEI. Low levels of fecal elastase (<200 µg/g stool, although even lower levels are more specific) or serum trypsin (<20 ng/mL) are usually observed in patients with PEI ^79– 82^. If PEI is suspected, obtaining fecal elastase and trypsin levels can confirm the diagnosis. It should be noted that, overall, serum trypsinogen is insensitive as a diagnostic test, with a sensitivity in the range of 33–65% ^79– 81^. However, a very low level of serum trypsin is a marker of severely compromised pancreatic function, and false positive results do not occur in patients with non-pancreatic steatorrhea ^82^. This makes trypsin an attractive screening test in patients with steatorrhea of pancreatic origin. A 72-hour analysis of fecal fat content on a high-fat diet is necessary to confirm steatorrhea and diagnose PEI, but this test is cumbersome and rarely performed.

Cornerstones in the treatment of PEI are PERT, support to cease smoking and alcohol consumption, consultation with a dietitian, and a systematic follow-up to assure optimal treatment effect ^70^. Treatment is aimed at the normalization of digestion, alleviation of PEI-linked symptoms, and prevention of morbidity and mortality associated with malnutrition as well as disease progression ^83^. To guarantee optimal efficacy of oral PERT, it is necessary to ensure proper administration, dose, and adjuvant therapy. Currently available enzyme products are mainly enteric-coated capsules and are identified by the amount of lipase (USP units) they contain (please refer to Table 1). Capsules should be administered with meals (as opposed to before or after) for optimal effect ^84^. The normal pancreas produces at least 90,000 USP units of lipase with each meal. The starting dose for PERT should be at least 40,000 to 50,000 USP units of lipase with each meal and half that amount with snacks ^39^. If signs or symptoms of maldigestion persist, the PERT dose can be increased up to 90,000 USP units of lipase (10% of normal output) with each meal ^39^, and proton pump inhibitors can be added, since bicarbonate secretion is impaired in CP. The addition of proton pump inhibitors ensures that the lipase is protected from denaturation by gastric acid, as pancreatic lipase has been shown to be irreversibly inactivated at a pH below 4 ^85^. Acid suppression is required if the non-enteric-coated preparation is used. If PERT is ineffective despite these measures, possible co-existing and/or alternative reasons for maldigestion such as small intestinal bacterial overgrowth should be investigated ^83^. A low-fat diet is no longer recommended to reduce steatorrhea because of the risk of exacerbating PEI-related weight loss and deficiencies of lipid-soluble vitamins ^86, 87^.

**Table 1.** Enzyme therapy for exocrine insufficiency.

Product	Formulation	Lipase content/capsule or pill
Zenpep®	Enteric-coated porcine	3,000, 5,000, 10,000, 15,000, 20,000, 25,000, 40,000
Creon®	Enteric-coated porcine	3,000, 6,000, 12,000, 24,000, 36,000
Pancreaze®	Enteric-coated porcine	4,200, 10,500, 16,800, 21,000
Pertzye®	Enteric-coated porcine with bicarbonate	4,000, 8,000, 16,000
Viokace®	Non-enteric-coated porcine tablet *	10,440, 20,880

*Needs to be co-administered with an H2 blocker or proton pump inhibitor

### Management of endocrine insufficiency

Diabetes has been recognized as a secondary complication of various pancreatic disorders such as AP and CP as well as pancreatic cancer ^88^. Diabetes secondary to pancreatic disease is commonly referred to as pancreatogenic diabetes or type 3c diabetes mellitus (DM). More than half of all patients with CP develop DM ^89^ due to the loss of complete islet cell mass, not just beta cells as in type 1 DM, or due to insulin resistance as in type 2 DM. The diagnosis of diabetes in CP patients relies on the same criteria as for all forms of diabetes: fasting plasma glucose level ≥126 mg/dL, 2-hour oral glucose tolerance test result >200 mg/dL, or hemoglobin A1c ≥6.5%. It is reasonable to repeat these tests on a yearly basis.

A unique characteristic of patients with type 3c diabetes is that they lose counter-regulatory hormones, such as glucagon and pancreatic polypeptide, and are more susceptible to hypoglycemia ^90^. Type 3c diabetes also puts patients at a particularly high risk of developing secondary pancreatic carcinoma ^2^. The use of an insulin-sensitizing agent such as metformin may reduce the risk of cancer in these patients ^39^. Associated malabsorption due to PEI often releases higher levels of gut hormones including GLP-1; therefore, the effectiveness of insulin secretagogues and incretin drugs is very low ^91, 92^. Treatment requires PERT to maximize incretin secretion and nutritional status in addition to diabetic medications and early referral to an endocrinologist for this brittle form of diabetes ^30, 90^.

## Conclusion

The etiology of CP has traditionally been classified as alcohol, hereditary, obstructive, hyperlipidemia, and idiopathic. Data indicate that AP progresses to RAP then to CP in a disease continuum. However, not all AP becomes recurrent, and not all RAP progresses to CP. Whether AP proceeds to RAP and to CP is determined by a multitude of risk factors, including exposure to alcohol, smoking, hereditary mutations, ductal obstruction, and autoimmune factors. Increased knowledge regarding these etiologies has enhanced our understanding of the disease and changed our approach to the diagnosis and management of this elusive disease. Current management of CP involves patient education, counseling regarding alcohol and tobacco abstinence, a multidisciplinary team approach to pain management, medical treatment of PEI, addressing malnutrition and osteoporosis, and adjustment of PERT and diabetic agents. In a carefully selected subset of patients, endoscopic and surgical intervention may be appropriate.
